# Safety and Efficacy of High-Dose Chemotherapy with TreoMel 200 vs. TreoMel 140 in Acute Myeloid Leukemia Patients Undergoing Autologous Stem Cell Transplantation

**DOI:** 10.3390/cancers16101887

**Published:** 2024-05-15

**Authors:** Matthias Eggimann, Dilara Akhoundova, Henning Nilius, Michèle Hoffmann, Michael Hayoz, Yolanda Aebi, Carlo R. Largiadèr, Michael Daskalakis, Ulrike Bacher, Thomas Pabst

**Affiliations:** 1 Department of Medical Oncology, Inselspital—Bern University Hospital, 3010 Bern, Switzerland; matthias.eggimann@students.unibe.ch (M.E.); dilara.akhoundovasanoyan@insel.ch (D.A.); michele.hoffmann@insel.ch (M.H.); 2 Department of Clinical Chemistry, Inselspital—Bern University Hospital, 3010 Bern, Switzerland; henning.nilius@insel.ch (H.N.); michael.hayoz@insel.ch (M.H.); yolanda.aebi@insel.ch (Y.A.); carlo.largiader@insel.ch (C.R.L.); 3 Central Hematology Laboratory, Department of Hematology, Inselspital—Bern University Hospital, University of Bern, 3012 Bern, Switzerland; michael.daskalakis@insel.ch (M.D.); veraulrike.bacher@insel.ch (U.B.)

**Keywords:** high-dose chemotherapy (HDCT), treosulfan and melphalan (TreoMel), acute myeloid leukemia (AML), progression-free survival (PFS), overall survival (OS), side effects, safety

## Abstract

**Simple Summary:**

Treosulfan and melphalan (TreoMel)-based high-dose chemotherapy (HDCT) has been successfully used as a conditioning regimen in acute myeloid leukemia (AML) patients undergoing autologous stem cell transplantation (ASCT). However, despite intensive first-line induction treatment and upfront consolidation with HDCT and ASCT, AML relapse rates are still high, and further efforts are needed to improve patient outcomes. In this study, we investigated how increased doses of melphalan impact the safety of HDCT with TreoMel and patient outcomes. A total of 51 AML patients were included in the analysis: 31 (60.8%) received standard-dose treosulfan combined with melphalan 140 mg/m^2^ (TreoMel 140) and 20 (39.2%) received melphalan 200 mg/m^2^ (TreoMel 200). There were no statistically significant differences in relapse (0.45 vs. 0.30, *p* = 0.381) and mortality rates (0.42 vs. 0.15, *p* = 0.064) between the melphalan 140 mg/m^2^ and 200 mg/m^2^ cohorts, nor for PFS (HR: 0.81, 95% CI: 0.29–2.28, *p* = 0.70) or OS (HR: 0.70, 95% CI: 0.19–2.57, *p* = 0.59) for the TreoMel 140 vs. TreoMel 200 cohorts. The side effect profile was comparable between both patient groups. Our results show that a higher melphalan dose is well tolerated. No significant differences for patient outcomes could be observed, possibly due to the relatively small patient cohort and short follow-up. Longer follow-up and prospective randomized studies would be required to confirm the safety profile and clinical benefit.

**Abstract:**

(1) Background: Treosulfan and melphalan (TreoMel)-based high-dose chemotherapy (HDCT) has shown promising safety and efficacy as a conditioning regimen for acute myeloid leukemia (AML) patients undergoing autologous stem cell transplantation (ASCT). However, despite intensive first-line induction treatment and upfront consolidation with HDCT and ASCT, AML relapse rates are still high, and further efforts are needed to improve patient outcomes. The aim of this study was to compare two melphalan dose schedules in regard to the safety of TreoMel HDCT and patient outcomes. (2) Methods: We retrospectively analyzed the safety and efficacy of two melphalan dose schedules combined with standard-dose treosulfan in AML patients undergoing HDCT and ASCT at the University Hospital of Bern, Switzerland, between August 2019 and August 2023. Patients received treosulfan 42 g/m^2^ combined with either melphalan 140 mg/m^2^ (TreoMel 140) or melphalan 200 mg/m^2^ (TreoMel 200). Co-primary endpoints were progression-free survival (PFS), overall survival (OS), as well as safety profile. (3) Results: We included a total of 51 AML patients: 31 (60.8%) received TreoMel 140 and 20 (39.2%) TreoMel 200. The patients’ basal characteristics were comparable between both cohorts. No significant differences in the duration of hospitalization or the adverse event profile were identified. There were no statistically significant differences in relapse (0.45 vs. 0.30, *p* = 0.381) and mortality rates (0.42 vs. 0.15, *p* = 0.064) between the melphalan 140 mg/m^2^ and 200 mg/m^2^ cohorts, nor for PFS (HR: 0.81, 95% CI: 0.29–2.28, *p* = 0.70) or OS (HR: 0.70, 95% CI: 0.19–2.57, *p* = 0.59) for the TreoMel 140 vs. TreoMel 200 cohort. (4) Conclusions: A higher dose of melphalan (TreoMel 200) was well tolerated overall. No statistically significant differences for patient outcomes could be observed, possibly due to the relatively small patient cohort and the short follow-up. A longer follow-up and prospective randomized studies would be required to confirm the safety profile and clinical benefit.

## 1. Introduction

Acute myeloid leukemia (AML) is a highly aggressive malignancy of clonal myeloid progenitor cells. Accounting for 1.0% of all new cancer cases and 1.9% of cancer deaths, AML is the second most common form of leukemia, with an incidence of 4.1 per 100,000 person-years [1]. Disease symptoms are due to rapid bone marrow failure as well as organ infiltration. Without treatment, AML is inevitably lethal [2,3,4]. The average age at diagnosis is 65 years, with >2/3 of cases presenting in patients above the age of 50 [5]. Despite relevant advances in systemic treatment options, 5-year survival rates are still poor. According to the SEER database, the 5-year survival rate was 31% [1]. Notably, individual patient outcomes are highly variable, depending on disease molecular features as well as the patient’s general condition and comorbidities [6].

Optimal risk assessment and prompt initiation of systemic therapy are essential to optimize patient outcomes. Risk assessment following the European LeukemiaNet (ELN) criteria is based on underlying genomic alterations [3]. Additionally, clinical factors relevant for optimal risk stratification include initial leukocyte count, AML subtype, and patient age and comorbidities [7,8]. Intensive induction and consolidation therapy is the strategy of choice in the curative setting, and the main goal of induction therapy is to achieve complete hematologic remission. In patients fit for intensive chemotherapy, an idarubicin/daunorubicin +/− cytarabine-based induction chemotherapy, combined with molecularly targeted agents, is the current standard of care. Induction chemotherapy is followed by stem cell mobilization and apheresis before initiation of consolidation therapy, which aims to prevent relapse. Based on risk stratification, distinct consolidation strategies may be used. Following the ELN guidelines, high-dose chemotherapy (HDCT) followed by autologous stem cell transplantation (ASCT) is a valid consolidation alternative for patients with a favorable risk profile, as well as for selected patients with intermediate risk, who either achieved negative minimal residual disease (MRD) after induction with 2–4 cycles of idarubicin and cytarabine (IDAC) or are unfit for allogenic HSCT [3,9]. In patients with adverse risk features, or who are intermediate risk with a low Hematopoietic Cell Transplantation-specific Comorbidity Index (HCT-CI) score, allogeneic stem cell transplantation should be considered whenever possible [9,10,11,12,13,14].

Treatment consolidation with HDCT with TreoMel has shown promising safety and efficacy data in AML patients undergoing ASCT [15]. For instance, in previous work, we showed that TreoMel has comparable efficacy and a more favorable toxicity profile, including lower rates of neurotoxicity and irreversible alopecia, as compared to busulfan and melphalan (BuMel) in this setting [15]. Based on these data, TreoMel has been adopted as the new standard-of-care HDCT prior to ASCT at our institution. However, further optimization of HDCT conditioning regimens is still needed. In this study, we compared the safety and efficacy of two melphalan dose schedules combined with standard-dose treosulfan: melphalan 140 mg/m^2^ (TreoMel 140) vs. 200 mg/m^2^ (TreoMel 200) [15,16,17,18,19,20,21,22,23,24]. While the TreoMel regimen has been commonly used with melphalan dosed at 140 mg/m^2^, given the adverse prognosis of AML, we decided to escalate the dosing to 200 mg/m^2^ for the patients treated from September 2021. Moreover, since relevant variability in treosulfan pharmacokinetics has been shown in previous studies, with potential impact on HSCT outcome and toxicity, we assessed treosulfan pharmacokinetics in both treatment cohorts [15,16,25,26].

## 2. Materials and Methods

### 2.1. Patient Cohort

This retrospective analysis was conducted at the University Hospital of Bern, Switzerland. Patients diagnosed with AML, aged 18 or older, receiving HDCT with TreoMel between August 2019 and August 2023 were included in this study. Patients were eligible for ASCT if they were classified either within the ELN favorable risk group or within the intermediate group and achieved MRD negativity during induction treatment. Additionally, patients with poor risk were included in case of a lack of an available donor or a refusal of allogenic stem cell transplantation. Patients were stratified into two cohorts according to the melphalan dose received: 140 mg/m^2^ (TreoMel 140) or 200 mg/m^2^ (TreoMel 200). Treosulfan was administered at a standard cumulative dose of 42 g/m^2^. Due to institutional guideline changes, all patients treated prior to September 2021 received TreoMel 140, while patients treated thereafter received TreoMel 200.

### 2.2. Clinical Procedures

Within the TreoMel 140 schedule, treosulfan was administered at 14 g/m^2^/day on days −4 to −2, followed by melphalan 140 mg/m^2^ on day −1, prior to ASCT (day 0). TreoMel 200 was administered as treosulfan 14 g/m^2^/day on days −5 to −3, followed by melphalan 100 mg/m^2^ on days −2 and −1, or as treosulfan 14 g/m^2^/day on days −4 to −2, followed by single-dose melphalan 200 mg/m^2^ on day −1. Following institutional guidelines, all patients received supportive therapy with G-CSF, as well as the following prophylaxis: sulfamethoxazole–trimethoprim, anti-fungal and antiviral, as well as tumor lysis and engraftment syndrome prophylaxis. To assess treosulfan’s pharmacokinetics, six peripheral blood samples were collected per patient: before and 30, 60, 120, 240, and 360 min after treosulfan infusion.

### 2.3. Study Endpoints and Data Collection

The co-primary endpoints were progression-free survival (PFS), overall survival (OS), and side effects during hospitalization. Secondary endpoints included relapse and mortality rates, as well as time to hematologic recovery and treosulfan pharmacokinetics. PFS was defined as time from HDCT to progression or death of any cause, and OS as time from HDCT to death. For safety assessment, the following side effects were registered: epileptic seizures, nausea, diarrhea, fever, infections, thrush, mucositis, headache, and fatigue. To assess how melphalan dose affects hematologic recovery after ASCT, we examined the times to neutrophil (>0.5 G/L) and platelet (>20 G/L) recovery. Laboratory data, including complete blood count and lactate dehydrogenase (LDH) values, were collected at diagnosis and prior to induction and HDCT. In addition, treosulfan plasma levels were monitored following a previously established pipeline [15]. Briefly, treosulfan plasma concentrations were determined with ultra-high-performance liquid chromatography–tandem mass spectrometry (UPLC-MS-MS). Mass spectrometric measurements were performed on a Xevo TQ-S (Waters Corp., Milford, MA, USA) using multiple reaction monitoring (MRM). Immediately after collection, blood samples were stabilized by the addition of a sodium citrate buffer and stored at −80 °C until analyses were performed. Six calibrator spiking solutions were prepared by diluting the stock solutions with methanol to final concentrations of 2.8, 5.6, 11.3, 22.5, 45, and 90 mg/L for treosulfan. An amount of 0.5 μL of the prepared samples was injected into a reversed-phase CORECTS UPLC T3 column and dissolved therein for 3.0 min. Electrospray ionization was used to introduce the eluent into the mass spectrometer (Xevo TQ-S, Waters Corp., Milford, MA, USA), which operated in positive-ion electrospray ionization (ESI +) mode. Data analysis was performed using TargetLynx (MassLynx software, version 4.1, Waters Corp., Milford, MA, USA), by comparing the area under the specific chromatograms of the MRM and the area of the isotope-labeled analog [15,25,27].

### 2.4. Statistical Analyses

Microsoft Excel, Graphpad Prism 8 for Windows (GraphPad Software, San Diego, CA, USA), and R (version 4.3.1, 2023) were used for statistical analyses. Excel was used to calculate the means and standard deviations. The normality of data distribution was assessed by the Shapiro–Wilk normality test. An unpaired *t*-test was used for normally distributed data. For non-normally distributed data, the Mann–Whitney nonparametric test was used. For categorical variables, chi-square and Fisher’s exact tests were used. *p*-values were calculated with Graphpad Prism and R. *p*-values were considered significant <0.05. PFS and OS were calculated with the Kaplan–Meier method. In addition, a Fine–Gray model was fitted to the data to compare the competing risks of death and recurrence in the different treatment groups. The cutoff date for data collection was set to 31 August 2023.

## 3. Results

### 3.1. Patient Basal Characteristics

Fifty-one AML patients undergoing HDCT and ASCT were included in the study: twenty (39.2%) patients received TreoMel 200, and thirty-one (60.8%) TreoMel 140. The patients’ basal characteristics were well balanced and are summarized in Table 1. While the median age was similar between both groups (51 vs. 56 years in the TreoMel 200 vs. TreoMel 140 cohorts, respectively), the male to female ratio differed significantly (0.67 vs. 2.44, *p* = 0.042). Blood counts at diagnosis were similar between both patient cohorts, and basal LDH values were numerically lower in the TreoMel 200 cohort: 646 vs. 940 U/L. The majority of patients had a favorable (55 vs. 32%, *p* = 0.187) or intermediate (45 vs. 39%, *p* = 0.877) risk following the ELN risk classification. The most common molecular alterations are summarized in Table 1.

### 3.2. Adverse Events during Hospitalization Post HDCT

The main adverse events during hospitalization post HDCT are summarized in Table 2. All but one patient within the TreoMel 140 group presented febrile episodes (98%). The infection rate was numerically lower in the TreoMel 200 cohort: 74% vs. 83% (*p*-value = 0.502). Infections were most frequently non-severe, and infectious agents included bacteria (e.g., streptococci, staphylococci, clostridia) and viruses (e.g., rhinoviruses and SARS-CoV-2). However, three (5.9%) patients died due to infectious complications. All but three patients in the TreoMel 140 cohort presented diarrhea (10.7%). Grade 3 diarrhea was more frequent in the TreoMel 200 (35%) group (*p*-value = 0.036). Only one patient presented grade 4 diarrhea. Fatigue (100% and 90.3%) and nausea (90% and 80.7%) were presented in the majority of patients, with numerically higher frequencies in the TreoMel 200 cohort. Headache occurred in about a quarter of patients. Both mucositis and thrush were numerically more frequent in the TreoMel 200 cohort. Overall, a similar adverse event profile was observed for both patient cohorts, with no significant differences except for grade 3 diarrhea.

### 3.3. Hematologic Recovery and Clinical Outcomes

Patients were transplanted with an average of 3.98 × 10^6^ autologous stem cells/kg body weight. Similar average counts of stem cells were transplanted in both patient cohorts. Patients within the TreoMel 200 cohort received an average of 4.23 × 10^6^/kg CD34+ cells vs. 3.81 × 10^6^/kg within the TreoMel 140 cohort (*p* = 0.867). Hematologic recovery times were shorter overall in the TreoMel 200 cohort. Neutrophil engraftment (defined as an absolute neutrophil count greater than 0.5 × 10^9^/L) was achieved after an average of 12.5 days in the TreoMel 200 group and of 15 days in the TreoMel 140 group (*p* = 0.084). The median thromboyte recovery (>20 × 10^9^/L) time was 16 vs. 19 days (*p* = 0.428). No differences in the duration of hospitalization were observed. Since the use of TreoMel 200 at our institution started from September 2021, the follow-up time was shorter for the TreoMel 200 cohort: 9 vs. 23 months. The relapse rate (30% vs. 45%, *p* = 0.381) and mortality rate (15% vs. 42%, *p*-value = 0.064) were numerically lower in the TreoMel 200 cohort; however, no statistically significant differences could be detected (Table 3).

### 3.4. Progression-Free and Overall Survival

**Figure 1:**
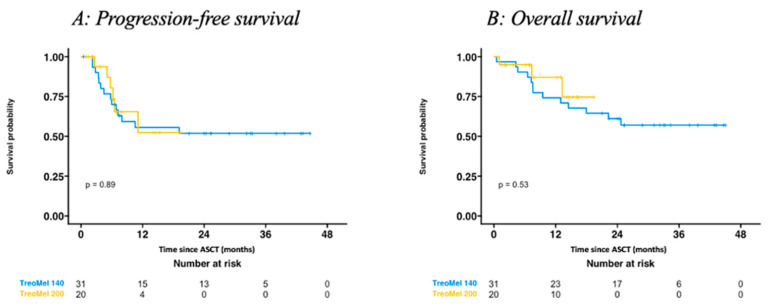
(**A**) Progression-free survival, and (**B**) overall survival of AML patients treated with TreoMel 140 vs TreoMel 200, followed by ASCT.

Figure 1A,B illustrate the PFS (*p* = 0.89) and OS (*p* = 0.53) times for patients treated with TreoMel 140 vs. TreoMel 200. Neither the median PFS nor OS were reached. The HR for PFS was 0.81 (95% CI: 0.29, 2.28) (*p* = 0.70) and for OS 0.70 (95% CI: 0.19, 2.57) (*p* = 0.59) for the TreoMel 140 vs. TreoMel 200 cohorts. Within the TreoMel 140 cohort, 13 (42%) patients deceased, with the following death causes: 10 AML-related, 1 infection-related, and 2 unclear. Within the TreoMel 200 cohort, in total three (15%) patients deceased: two due to infection and one due to an unclear cause. For both PFS and OS, no significant differences between both treatment cohorts were observed at data cut-off. However, the shorter follow-up time of the TreoMel 200 cohort should be taken into consideration.

### 3.5. Univariate and Multivariate Cox Proportional-Hazard Models for Relapse and Mortality

Results of the univariate and multivariate Cox proportional hazard models for AML relapse and mortality are summarized in Table 4 and Table 5. The results of the Fine–Gray competing risk regression for AML relapse and survival are summarized in Table 6. The following variables were included in the analyses: melphalan dose, age, sex, hemoglobin, leukocytes, platelets, blasts in peripheral blood, blasts in bone marrow, LDH at diagnosis, number of induction cycles, and number of transplanted stem cells. In the multivariate analysis, a higher dose of melphalan was associated with an HR of 0.62 for AML relapse and an HR of 0.26 for the risk of death (*p* = 0.60 and *p* = 0.15, respectively).

### 3.6. Treosulfan Plasma Concentrations

**Figure 2:**
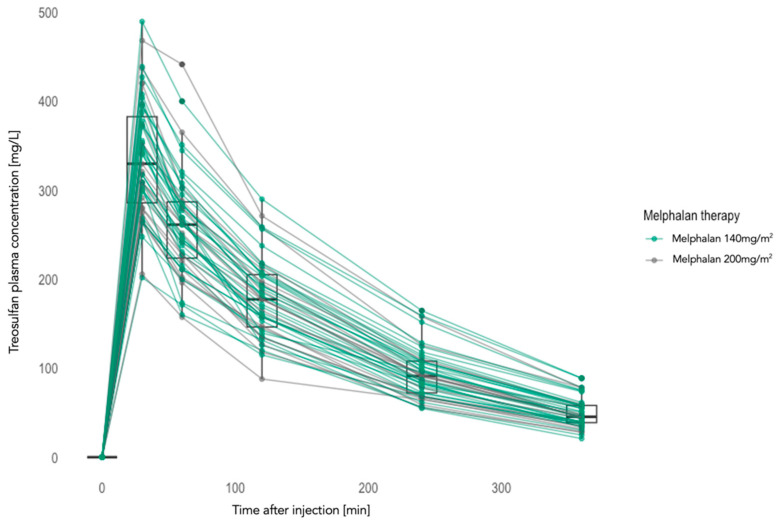
Mean treosulfan plasma concentrations over time (TreoMel 200, n = 200; TreoMel 140, n = 31).

In order to assess how melphalan dose modification (140 vs. 200 mg/m^2^) impacts treosulfan pharmacokinetics, we measured the plasma concentrations of treosulfan previous to treosulfan infusion (T0) and at 30 (T30), 60 (T60), 120 (T120), 240 (T240), and 360 (T360) minutes post treosulfan infusion. Numerically slightly higher treosulfan concentrations were detected in the TreoMel 140 cohort. However, no statistical differences were observed, as shown in Table 7 and Figure 2. Moreover, no differences in treosulfan areas under the curve (AUCs) were detected between both treatment cohorts: 805.63 ± 207.28 mg/L*h for the TreoMel 200 cohort and 852.67 ± 199.37 mg/L*h for the TreoMel 140 cohort (*p*-value = 0.4328).

## 4. Discussion

Extensive efforts have been made over recent decades to improve the efficacy and safety of pre-ASCT HDCT regimens in AML [28,29,30]. In a large study from the AML Working Party of the European Society for Bone and Marrow Transplantation (EBMT), BuMel showed a lower relapse rate, an improved leukemia-free survival and better OS at 5 years in patients with adverse risk factors, as compared to busulfan and cyclophosphamide (BUCY)-based HDCT [30]. Further pre-ASCT conditioning regimens, such as fludarabine-based regimens, have been more commonly used in the context of non-Hodgkin lymphoma [31,32,33]. In previous work, we showed that a treosulfan and melphalan-based regimen (TreoMel) has promising activity in AML patients with a more favorable safety profile as compared to BuMel [15,30]. In contrast to BuMel, we observed no cases of irreversible alopecia following conditioning with TreoMel [15]. Moreover, we reported a lower incidence of central neurotoxicity since treosulfan and its biologically active metabolites (epoxides) are unable to cross the blood–brain barrier [15]. This low rate of central neurotoxicity has also previously been shown in preclinical models [34,35,36]. Additionally, treosulfan has been correlated with a lower frequency of early pulmonary and liver toxicity, as compared with busulfan-based conditioning regimens [37]. Based on these data, TreoMel has been adopted as standard conditioning regimen for AML patients at our institution. However, despite more effective and less toxic induction and consolidation regimens, relapse rates and mortality in AML patients remain high, so further optimization of AML first-line treatment is needed. In another study from our group, by Gillich et al. [25], we showed that conditioning with TreoMel 200 is highly effective and feasible in fit multiple myeloma patients. From September 2021, we modified our institutional HDCT standard for AML patients from TreoMel 140 to TreoMel 200 by increasing the melphalan dose from 140 mg/m^2^ to 200 mg/m^2^ [15]. In the current study, we investigated how this dose modification impacted treatment toxicity and patient outcomes [37,38,39,40]. Our current study was not able to show statistically significant differences in PFS or OS between the two patient cohorts, treated with TreoMel 140 and TreoMel 200, respectively. Relapse and mortality rates were numerically lower in the melphalan 200 mg/m^2^ cohort; however, the correlation with melphalan dose also resulted non-significant in the multivariate Cox analysis. We observed no significant differences in acute toxicity or hospitalization duration between both treatment cohorts. Moreover, hematologic recovery occurred more rapidly in the TreoMel 200 cohort. In line with the safety profile observed by Gurevich et al., TreoMel was well tolerated, regardless of the employed melphalan dose [15]. Relevantly, no neurotoxicity in terms of epileptic seizures was observed. However, three (5.9%) patients still died due to infectious complications, underlining the potential risks of HDCT [34,35,36]. Overall, the patients’ basal characteristics were well balanced between both treatment cohorts, except for gender distribution and the ELN adverse risk subgroup. However, the disbalance in the ELN adverse risk subgroup only concerned a small proportion of our patient population. Some limitations of our study are the relatively small patient cohort; the short follow-up, especially for the TreoMel 200 cohort (since this cohort is more recent in time), with potential impact on the relapse rate and survival endpoint results; as well as the retrospective, single-center design of the study. A longer follow-up and a larger patient cohort would be needed to detect potential differences in survival outcomes. Additionally, the retrospective design of the study might lead to patient selection bias. Moreover, future studies should integrate further variables, such as specific molecular profiles or disease responses previous to HSCT, as well as analyze a broader spectrum of adverse events.

## 5. Conclusions

In summary, our study showed that HDCT with melphalan 200 mg/m^2^ and treosulfan 42 g/m^2^ (TreoMel 200) was well tolerated overall. No significant differences for patient outcomes could be observed, possibly due to the relatively small patient cohort and short follow-up. Longer follow-up and prospective randomized studies would be required to confirm the safety and efficacy of TreoMel 200.

## Figures and Tables

**Table 1:** Patient characteristics at diagnosis.

Characteristics	TreoMel 200 (n = 20)	TreoMel 140 (n = 31)	All Patients (n = 51)	*p*-Value
Mean age at diagnosis, years (range)	51.25 (17–74)	55.95 (33–73)	54.10 (17–74)	0.226
Males/females (ratio)	8/12 (0.67)	22/9 (2.44)	30/21 (1.43)	**0.042**
ELN risk classification favorable, n (%)	11 (55)	10 (32)	21 (41)	0.187
ELN risk classification intermediate, n (%)	9 (45)	12 (39)	21 (41)	0.877
ELN risk classification adverse, n (%)	0 (0)	9 (29)	9 (18)	**0.008**
Most common molecular genetic abnormalities *				
NPM1mut, n (%)	13 (65)	15 (48)	28 (55)	0.381
FLT3-ITD/TKD, n (%)	4 (20)	12 (39)	16 (31)	0.221
IDH1mut, n (%)	1 (5)	4 (13)	5 (10)	0.636
IDH2mut, n (%)	4 (20)	9 (29)	13 (25)	0.529
RUNX1-RUNX1T1, n (%)	1 (5)	2 (6)	3 (6)	>0.9999
CBFB-MYH11, n (%)	1 (5)	2 (6)	3 (6)	>0.9999
Mean Peripheral Blood Parameters				
WBC, G/L (±SD)	42.67 (±45.45)	43.64 (±71.31)	43.26 (±61.94)	0.321
Platelets, G/L (±SD)	75.90 (±44.59)	86.49 (±56.34)	82.34 (±51.84)	0.482
Hemoglobin, g/L (±SD)	87.85 (±21.23)	92.58 (±21.16)	90.73 (±21.10)	0.440
Peripheral blasts, % (±SD)	42.29 (±36.54)	43.82 (±29.81)	43.76 (±32.26)	0.875
BM blasts, % (±SD)	74.25 (±20.66)	73.95 (±24.31)	74.07 (±22.74)	0.768
LDH, U/L (±SD)	646.35 (±519.36)	940.48 (±936.15)	825.14 (±805.83)	0.276

TreoMel 200: treosulfan + melphalan 200 mg/m^2^ patient cohort, TreoMel 140: treosulfan + melphalan 140 mg/m^2^ patient cohort, BM: bone marrow, LDH: lactate dehydrogenase, ±SD: standard deviation, WBC: white blood cell, * some patients had more than one abnormality.

**Table 2:** Adverse events during hospitalisation post HDCT.

Toxicity, n (%)	TreoMel 200 (n = 20)	TreoMel 140 (n = 31)	All Patients (n = 51)	*p*-Value
Febrile episode	20 (100.0)	30 (96.8)	50 (98.0)	>0.9999
Infection	14 (73.7)	25 (83.3)	39 (79.6)	0.502
Diarrhea	20 (100.0)	28 (90.3)	48 (94.1)	0.271
Grade 1	8 (40.0)	18 (64.3)	26 (51.0)	0.258
Grade 2	5 (25.0)	6 (21.4)	11 (21.6)	0.733
Grade 3	7 (35.0)	3 (10.7)	10 (19.6)	**0.036**
Grade 4	0 (0.0)	1 (3.6)	1 (2.0)	>0.9999
Grade 5	0 (0.0)	0 (0.0)	0 (0.0)	>0.9999
Nausea	18 (90.0)	25 (80.7)	43 (84.3)	0.456
Mucositis	12 (60.0)	14 (45.2)	26 (51.0)	0.393
Headache	5 (25.0)	7 (22.6)	12 (23.5)	>0.9999
Thrush	4 (20.0)	4 (12.9)	8 (15.7)	0.696
Epileptic seizure	0 (0.0)	0 (0.0)	0 (0.0)	>0.9999
Fatigue	20 (100.0)	28 (90.3)	48 (94.1)	0.271

TreoMel 200: treosulfan + melphalan 200 mg/m^2^ patient cohort, TreoMel 140: treosulfan + melphalan 140 mg/m^2^ patient cohort, Grade 1: (<4 stools/d above baseline), Grade 2: (4–6 stools/d above baseline), Grade 3: (≥7 stools/d above baseline; stool incontinence, hospitalization indicated; limited activities of daily living), Grade 4: (life-threatening consequences, urgent intervention indicated), Grade 5: (Death).

**Table 3:** Details of engraftment and clinical outcomes.

Parameters	TreoMel 200 (n = 20)	TreoMel 140 (n = 31)	All Patients (n = 51)	*p*-Value
Median follow up, months (range)	8.90 (1–19)	23.27 (0.5–45)	17.64 (0.5–45)	**<0.0001**
Median time from diagnosis to ASCT, days (range)	122.60 (84–223)	123.19 (87–205)	122.96 (84–223)	0.550
Median CD34^+^ cells at ASCT, n × 10^6^ /kg b.w. (range)	4.23 (2.18–11.04)	3.81 (0.96–10.84)	3.98 (0.96–11.04)	0.867
Median time to neutrophil recovery, days (range)	12.45 (9–19)	15.20 (10–36)	14.10 (10–36)	0.084
Median time to platelet recovery, days (range)	15.95 (6–52)	19.19 (8–66)	17.92 (6–66)	0.428
Median hospitalization duration, days (range)	25.55 (19–39)	23.40 (17–59)	24.26 (17–59)	0.130
Relapse, n (%)	6 (30.00)	14 (45.16)	20 (39.22)	0.381
Median interval to relapse, months (range)	6.17 (3–11)	6.07 (2–19)	6.10 (2–19)	0.709
Deaths, n (%)	3 (15.00)	13 (41.94)	16 (31.37)	0.064
Median time to death, months (range)	7.33 (1–14)	10.88 (0.5–25)	10.22 (0.5–25)	0.465

TreoMel 200: treosulfan + melphalan 200 mg/m^2^ patient cohort, TreoMel 140: treosulfan + melphalan 140 mg/m^2^ patient cohort.

**Table 4:** Univariate and multivariate Cox proportional-hazard models for the hazards of a recurrence of AML.

Parameters	Univ. HR	95% Cl	*p*-Value	Mult. HR *	95% Cl	*p*-Value
Melphalan 200 mg/m^2^	0.93	[0.35; 2.45]	0.88	0.68	[0.21; 2.19]	0.52
Age [per year]	0.99	[0.96; 1.03]	0.77	1.01	[0.97; 1.05]	0.70
Male sex	1.11	[0.44; 2.78]	0.83	1.55	[0.46; 5.21]	0.48
Hemoglobin [per g/L]	0.97	[0.95; 1.00]	**0.04**	0.98	[0.95; 1.01]	0.14
Leucocytes [per G/L]	1.01	[1.00; 1.01]	0.07	1.01	[1.00; 1.01]	0.22
Thrombocytes [per G/L]	0.99	[0.98; 1.00]	0.12	0.99	[0.98; 1.00]	0.30
Blasts in the peripheral blood [per %]	1.01	[1.00; 1.03]	0.09	1.00	[0.98; 1.03]	0.67
Blasts in the bone marrow [per %]	1.01	[0.99; 1.04]	0.21	1.01	[0.97; 1.04]	0.74
LDH [U/L]	1.00	[1.00; 1.00]	0.73	1.00	[1.00; 1.00]	0.27
Induction cycles	0.73	[0.36; 1.52]	0.40	0.72	[0.30; 1.75]	0.47
Amount of stemcells [per 10^6^/kgKG]	0.96	[0.79; 1.18]	0.73	0.94	[0.74; 1.19]	0.62

TreoMel 200: treosulfan + melphalan 200 mg/m^2^ patient cohort, TreoMel 140: treosulfan + melphalan 140 mg/m^2^ patient cohort, * Adjusted for all predictors. CI = confidence interval, HR = hazard ratio, Mult. = Multivariate, Univ = Univariate.

**Table 5:** Univariate and multivariate Cox proportional-hazard models for the hazards of death (all causes) (TreoMel 200 n = 20, TreoMel 140 n = 31).

Parameters	Univ. HR	95% Cl	*p*-Value	Mult. HR *	95% Cl	*p*-Value
Melphalan 200 mg/m^2^	0.66	[0.18; 2.41]	0.53	0.26	[0.04; 1.60]	0.15
Age [per year]	1.03	[0.98; 1.08]	0.20	1.04	[0.99; 1.10]	0.14
Male sex	1.06	[0.36; 3.07]	0.92	0.67	[0.14; 3.15]	0.62
Hemoglobin [per g/L]	0.98	[0.96; 1.01]	0.14	0.98	[0.94; 1.01]	0.22
Leucocytes [per G/L]	1.01	[1.00; 1.01]	0.15	1.01	[1.00; 1.02]	0.06
Thrombocytes [per G/L]	0.99	[0.98; 1.00]	0.09	0.99	[0.98; 1.00]	0.15
Blasts in the peripheral blood [per %]	1.01	[1.00; 1.03]	0.14	1.03	[0.99; 1.06]	0.11
Blasts in the bone marrow [per %]	1.00	[0.98; 1.02]	0.90	0.96	[0.92; 1.00]	**0.04**
LDH [U/L]	1.00	[1.00; 1.00]	0.58	1.00	[1.00; 1.00]	0.23
Induction cycles	1.11	[0.69; 1.80]	0.67	1.23	[0.59; 2.55]	0.58
Amount of stemcells [per 10^6^/kgKG]	1.05	[0.84; 1.32]	0.64	1.12	[0.87; 1.45]	0.38

TreoMel 200: treosulfan + melphalan 200 mg/m^2^ patient cohort, TreoMel 140: treosulfan + melphalan 140 mg/m^2^ patient cohort, * Adjusted for all predictors. CI = confidence interval, HR = hazard ratio, Mult. = Multivariate, Univ = Univariate.

**Table 6:** Multivariable Fine–Gray subdistribution hazard model for the competing risk between recurrence of AML and death.

	Recurrence			Survival		
	Mult. * HR	95% CI	*p*-Value	Mult. * HR	95% CI	*p*-Value
Highdose Melphalan	0.62	[0.10; 3.75]	0.6	0.3	[0.06; 1.49]	0.14
Age [per year]	0.99	[0.94; 1.05]	0.79	2.06	[1.57; 2.69]	<0.001
Male sex	2.64	[0.47; 14.8]	0.27	0.01	[0.54; 11.97]	0.006
Hemoglobin [per g/L]	0.97	[0.93; 1.02]	0.24	1.11	[1.05; 1.17]	<0.001
Leucocytes [per G/L]	1.01	[1.00; 1.02]	0.11	0.99	[0.97; 1.01]	0.45
Thrombocytes [per G/L]	1	[0.98; 1.01]	0.38	1.04	[1.00; 1.08]	0.04
Blasts in the peripheral blood [per %]	1.01	[1.00; 1.03]	0.83	1.02	[0.99; 1.05]	0.15
Blasts in the bone marrow [per %]	1	[0.97; 1.04]	0.89	1.07	[1.02; 1.11]	0.002
LDH [U/L]	1	[1.00; 1.00]	0.23	1	[1.00; 1.00]	0.98
Induction cycles	0.84	[0.43; 1.62]	0.59	0	[0.00; 0.00]	<0.001
Amount of stem cells [per 10^6^/kg BW]	1.05	[0.79; 1.40]	0.72	0.07	[0.03; 0.19]	<0.001
Treosulfan AUC	1	[1.00; 1.00]	0.43	1	[1.00; 1.00]	0.087
Peak Treosulfan concentration (mg/L)	1.01	[1.00; 1.03]	0.061	0.84	[0.73; 0.96]	0.009

* Adjusted for all predictors. CI = confidence interval, HR = hazard ratio, Mult. = Multivariate.

**Table 7:** Mean treosulfan plasma concentration over time.

Time after Injection in Minutes	TreoMel 200 (n = 20)	TreoMel 140 (n = 31)	All Patients (n = 51)	*p*-Value
T0 mg/L	<20	<20	<20	>0.9999
T30 mg/L	328.4	340.4	335.7	0.53
T60 mg/L	259.1	261.7	260.7	0.88
T120 mg/L	175.1	181.1	178.8	0.63
T240 mg/L	89.1	97.0	93.9	0.31
T360 mg/L	47.1	51.1	49.6	0.40

TreoMel 200: treosulfan + melphalan 200 mg/m^2^ patient cohort, TreoMel 140: treosulfan + melphalan 140 mg/m^2^ patient cohort.

## Data Availability

The data presented in this study are available in this article.
